# Targeted gene silencing of CCL2 inhibits triple negative breast cancer progression by blocking cancer stem cell renewal and M2 macrophage recruitment

**DOI:** 10.18632/oncotarget.9885

**Published:** 2016-06-07

**Authors:** Wei Bin Fang, Min Yao, Gage Brummer, Diana Acevedo, Nabil Alhakamy, Cory Berkland, Nikki Cheng

**Affiliations:** ^1^ Department of Pathology and Laboratory, University of Kansas Medical Center, Kansas City, KS 66160, USA; ^2^ Department of Pharmaceutical Chemistry, University of Kansas, Lawrence, KS 66045, USA

**Keywords:** CCL2, TAT cell penetrating peptide, breast cancer, macrophage, cancer stem cell

## Abstract

Triple negative breast cancers are an aggressive subtype of breast cancer, characterized by the lack of estrogen receptor, progesterone receptor and Her2 expression. Triple negative breast cancers are non-responsive to conventional anti-hormonal and Her2 targeted therapies, making it necessary to identify new molecular targets for therapy. The chemokine CCL2 is overexpressed in invasive breast cancers, and regulates breast cancer progression through multiple mechanisms. With few approaches to target CCL2 activity, its value as a therapeutic target is unclear. In these studies, we developed a novel gene silencing approach that involves complexing siRNAs to TAT cell penetrating peptides (Ca-TAT) through non-covalent calcium cross-linking. Ca-TAT/siRNA complexes penetrated 3D collagen cultures of breast cancer cells and inhibited CCL2 expression more effectively than conventional antibody neutralization. Ca-TAT/siRNA complexes targeting CCL2 were delivered to mice bearing MDA-MB-231 breast tumor xenografts. *In vivo* CCL2 gene silencing inhibited primary tumor growth and metastasis, associated with a reduction in cancer stem cell renewal and recruitment of M2 macrophages. These studies are the first to demonstrate that targeting CCL2 expression *in vivo* may be a viable therapeutic approach to treating triple negative breast cancer.

## INTRODUCTION

The molecular heterogeneity of breast cancers is well recognized, with at least five different subtypes identified through molecular profiling. Basal-like breast cancers comprise up to 15% of all of breast cancer cases diagnosed in the US and Canada. However, patients diagnosed with basal-like breast cancers exhibit the shortest time for overall survival and recurrence free survival [1-3]. 75% of basal–like breast cancers are triple negative (TNBC); i.e, they lack expression of Her2, progesterone receptor and estrogen receptors. TNBCs are non-responsive to endocrine and targeted Her2 therapies [4-6]. In order to treat TNBC more effectively, it is necessary to identify new molecular targets tailored to this disease subtype.

CCL2 (MCP-1) represents a promising molecular target for therapeutic intervention. CCL2 belongs to a family of small soluble molecules (8kDa) known as chemokines, and bind to G protein coupled receptors to regulate macrophage recruitment during wound healing, inflammation and infection [7-14]. Chronic overexpression of CCL2 contributes to inflammatory diseases including rheumatoid arthritis, fibrosis and macular degeneration [15, 16]. Overexpression of CCL2 in the tumor epithelium correlates with tumor grade and poor patient prognosis in various tumor types including: gliomas, prostate cancers, ovarian cancers and breast cancers [17, 18]. In primary prostate and mammary tumors, CCL2 overexpression correlates with recruitment of M2 polarized macrophages, a subpopulation of macrophages that facilitate tumor progression through secretion of growth and survival factors [19, 20]. In functional studies, CCL2 antibody neutralization in 4T1 or MDA-MB-231 breast tumor xenografts inhibited tumor growth and metastasis, while decreasing the levels of macrophages in the primary tumors, indicating that CCL2 promotes progression of TNBC [21-23] through macrophage dependent mechanisms. Recent studies demonstrate that CCL2 also signal to breast cancer cells to regulate survival and invasion [24]. These studies indicate that CCL2 regulates multiple mechanisms of breast cancer progression, demonstrating potential value as a therapeutic target.

Currently, the value of CCL2 as a therapeutic target is controversial. CCL2 neutralizing antibodies effectively blocked rheumatoid arthritis in mouse models, yet failed in clinical trials. Patients treated with CCL2 neutralizing antibodies showed accumulating levels of protein [25]. In breast tumor bearing mice, continuous treatment with CCL2 neutralizing antibodies inhibited mammary tumor progression. However, termination of treatment led to increased metastasis, which was associated with increased tumor angiogenesis [26]. These studies indicate that either CCL2 is not an effective therapeutic target or that neutralizing antibodies are a suboptimal method for blocking CCL2 activity. To more clearly evaluate these possibilities, we developed a novel approach to target CCL2 through gene silencing.

Gene silencing through small interfering RNAs (siRNAs) could selectively inactivate expression and activity of critical oncogenes, but would require a carrier to efficiently and specifically penetrate tumor cells and tissues [27-29]. The HIV-1 derived trans-activating transcriptor peptide (TAT_49-57_: RKKRRQRRR) exhibits unusual properties by efficiently penetrating cell membranes, independent of temperature or cell surface receptor expression but do not stably complex with siRNAs or DNA [30-32]. Recent discoveries made by our research group showed that calcium ions strengthened the interactions between TAT peptides to siRNA and plasmid DNA, through non-covalent cross-linking. Furthermore, calcium crosslinking of TAT (Ca-TAT) to siRNA or plasmid DNA condensed these complexes to nanoparticles, enhancing cellular transfection and gene knockdown more effectively, and with much lower toxicity than TAT CPPs alone or conventional polyethyleneimine particles [33-35].

Using Ca-TAT/siRNA complexes, we sought to evaluate the therapeutic efficacy of targeting gene expression of CCL2 in TNBC. Ca-TAT/siRNA complexes were observed to efficiently transfect MDA-MB-231 and DCIS.com breast cancer cells plated in 2D and 3D cultures, and reduce CCL2 expression more effectively than CCL2 neutralizing antibodies. In contrast to CCL2 antibody neutralization of breast tumor xenografts [21, 22, 26], CCL2 gene silencing did not increase tumor cell apoptosis or affect tumor angiogenesis. Instead, CCL2 gene silencing lead to increased tumor cell necrosis and autophagy, associated with a reduction in the number of cancer stem cells and recruitment of M2 macrophages. CCL2 gene silencing but not CCL2 antibody neutralization, significantly reduced CCL2 expression in breast cancer cells over time. Our studies are the first to demonstrate that targeting CCL2 expression exerts therapeutic effects different from blocking CCL2 activity through antibody binding. This study demonstrates a novel approach to target CCL2 and reveals important insight into the effects of CCL2 gene silencing on necrosis, autophagy, cancer stem cell renewal and macrophage recruitment.

## RESULTS

### Ca-TAT/siRNA particles target carcinoma cells to reduce CCL2 expression

**Figure 1:**
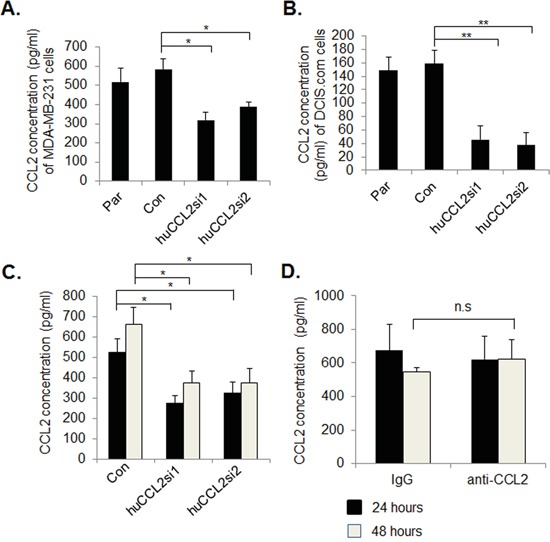
Ca-TAT peptides complexed to CCL2 siRNAs significantly reduce CCL2 protein expression in breast cancer cells **A.** MDA-MB-231 or **B**. DCIS.com cells cultured in 2D were transfected with Ca-TAT peptides complexed to control (Con) or CCL2 siRNAs (huCCL2si1 or huCCL2si2). 48 hours post transfection, conditioned media was measured by CCL2 ELISA. Par= Parental. **C-D.** MDA-MB-231cells were cultured in 3D collagen, and were transfected with Ca-TAT peptides complexed to siRNAs (C) or treated with 10 μg/ml anti-CCL2 or IgG (D). CCL2 expression was measured in conditioned medium by ELISA, 24 hours and 48 hours post-treatment. Statistical analysis was performed by One way ANOVA followed by Bonferonni post-hoc analysis. Statistical significance was determined by p-value less than 0.05. *p<0.05, **p<0.01, n.s=not significant. Mean+SEM is shown.

We first characterized the efficiency of gene silencing from Ca-TAT/siRNA complexes using cell culture models. In previous studies, we demonstrated that CaCl_2_ concentration and N/P ratio were significant factors affecting transfection efficiency of cultured PyVmT mammary carcinoma cells [36]. Using a luciferase reporter assay, we had determined that N/P=5 and 75 mM CaCl_2_ transfected mammary carcinoma cells most efficiently. Using this formula, Ca-TAT peptides were complexed to control eGFP siRNA, huCCL2si1 or huCCL2si2. According to ELISA analysis of 2D cultures, Ca-TAT delivery of huCCL2si1 or huCCL2si2 to either MDA-MB-231 or DCIS.com cells resulted in over a 50% knockdown in CCL2 expression, compared to Ca-TAT peptides complexed to control siRNA (Figure 1A-1B). We next determined the transfection efficiency of Ca-TAT/siRNA complexes in 3D collagen cultures, which closely model the architecture of breast tumor tissues [37-39]. Ca-TAT delivery of huCCL2si1 or huCCL2si2 efficiently transfected MDA-MB-231 breast cancer cells embedded in collagen and silenced CCL2 expression at 24 and 48 hours (Figure 1C). CCL2 neutralizing antibodies did not significantly affect CCL2 expression in 3D cultures (Figure 1D). In summary, these studies indicate that Ca-TAT/siRNA complexes efficiently knock down CCL2 expression in breast cancer cells.

### Inhibition of tumor growth and invasion with CCL2 gene silencing

**Figure 2:**
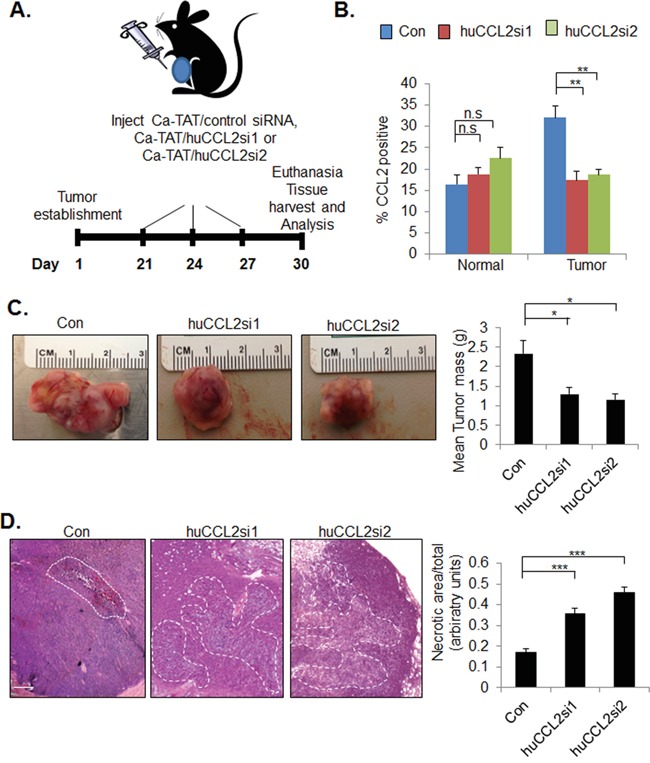
Ca-TAT delivery of CCL2 siRNAs inhibits growth and enhances cell death of primary MDA-MB-231 tumor xenografts **A.** Experimental plan for treatment of MDA-MB-231 tumor bearing mice with Ca-TAT/siRNA complexes. 21 days post transplantation, tumors received injections every three days with Ca-TAT peptides complexed to 10 micrograms control or CCL2 siRNAs (huCCL2si1, huCCL2si2) for a total of three injections, n=6 per group. Animals were euthanized 30 days post-transplantation for tissue harvest and analysis. **B.** Tumor and surrounding normal mammary tissues were analyzed for CCL2 expression by flow cytometry **C.** Representative tumors with quantified tumor mass. **D.** Representative H&E stain of primary tumor xenografts. Necrotic areas were identified on H&E stains at 5 different depths of the tumor and quantified by Image J analysis. Necrotic areas are outlined. Scale bar= 200 microns. Statistical analysis was performed by One way ANOVA followed by Bonferonni post-hoc comparisons with Control siRNA group. Statistical significance was determined by p-value less than 0.05. **p<0.01, ***p<0.001, n.s= not significant. Mean+SEM is shown.

To recapitulate formation of TNBC with a humanized stroma, MDA-MB-231 cells were co-transplanted with human carcinoma associated fibroblasts in collagen and transplanted in the inguinal mammary glands of nude mice [40, 41]. When tumors grew to approximately 0.5 cm in diameter, the xenografts received injections every three days with Ca-TAT complexed to control siRNA, huCCL2si1 or huCCL2si2, for a total of three injections per experimental group. Animals were sacrificed 30 days post-transplantation, when control tumors reached approximately 1.5 cm3 (Figure 2A), the maximum allowable tumor size, as outlined by the institutional animal care and use committee. CCL2 expression levels in tumor tissues or surrounding normal tissues were distinguished through flow cytometry staining with antibodies recognizing murine or human specific CCL2. Tumors receiving injections of Ca-TAT/huCCL2si1 or Ca-TAT/huCCL2si2 showed a significant 45% reduction in cells expressing overall human CCL2 protein compared to tumors receiving injections of control siRNA. Silencing of *CCL2* in tumor tissues down-regulated CCL2 protein expression to levels found in normal mammary tissues (Figure 2B). There were no significant differences in murine CCL2 protein expression with Ca-TAT delivery of control siRNA, huCCL2si1 or huCCL2si2, indicating that CCL2 expression in normal tissues were not affected by multiple injections of these complexes.

**Figure 3:**
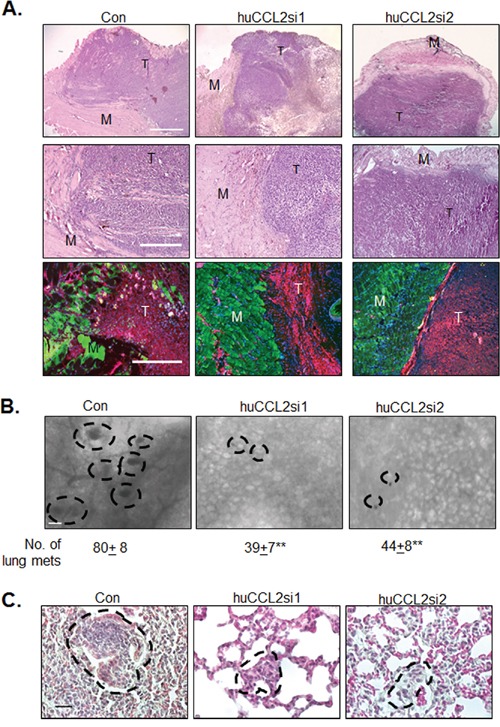
*CCL2* gene silencing inhibits primary and secondary invasion of MDA-MB-231 breast tumors **A.** Top panels: Low magnification (4X) H&E stain with muscle tissue (M) and tumor tissue (T). Scale bar=1000 microns. Middle panels: Higher magnification (10x) H&E stain with muscle tissue and tumor tissue. Scale bar=400 microns. Bottom panels: CO-IF stain of Calsquestrin (green) and CK5 (red) in primary tumor xenografts. Overlays of Calsequestrin, CK5 and DAPI stain are shown. Scale bar= 400 microns. **B.** Metastatic lesions throughout the lung tissue were visually identified by hematoxylin staining as round shaped nodules using an inverted microscope and quantified. Representative images are shown. Metastatic nodules are outlined. Scale bar=80 microns. **C.** H&E stain of lung tissues. Metastatic lesions are circled. Scale bar=40 microns. Statistical analysis was performed by One way ANOVA followed by Bonferonni post-hoc comparisons with Control siRNA group (Con). Statistical significance was determined by p-value less than 0.05. **p<0.01. Mean+SEM is shown. N=6 animals per group.

We determined the effects of CCL2 gene silencing on primary tumor growth and invasion. Compared to tumors treated with Ca-TAT/control siRNA complexes, tumors treated with Ca-TAT/huCCL2si1 exhibited a significant 40% decrease in mass, while tumors treated with Ca-TAT/huCCL2si2 exhibited a decrease in mass by 46% (Figure 2C). By H&E stain, tumor tissues treated with complexes of Ca-TAT/huCCL2si1 or Ca-TAT/huCCL2si2 showed exhibited extensive necrotic cell death throughout the tumor, as indicated by loss of tumor cell membrane and nuclear integrity (Figure 2D). Tumor tissues were examined for changes in invasion into muscle tissue. By H&E stain and by co-immunofluorescence staining for Calsequestrin and Cytokeratin 5 (CK5), control treated tumors showed extensive invasion into muscle tissue (Figure 3A). Tumors treated with Ca-TAT/huCCL2si1 or Ca-TAT/huCCL2si2 complexes showed a visible decrease in local invasion. In blinded studies, three different individuals scored tumor sections stained by H&E for invasion into muscle tissue. Sections at three different depths of the tumor were scored. By statistical analysis, we observed that tumors treated with Ca-TAT/huCCL2si1 or Ca-TAT/huCCL2si2 has a significant number of lower scores than the control group (Table 1). When we analyzed for changes in metastasis, we observed a significant decrease in the number and size of lung metastases in mice treated with Ca-TAT/huCCL2si1 or Ca-TAT/huCCL2si2 complexes (Figure 3B-3C). These data indicate that Ca-TAT delivery of CCL2 siRNAs inhibits breast tumor growth, invasion and metastasis.

**Table 1:** Scoring for muscle invasion at multiple tissue depths

	Experimental group	0 (No/little invasion)	1 (Some invasion)	2 (High invasion)	p-value
Individual 1	**Control**	5	8	5	0.04
	**huCCL2si1**	12	6	0	
	**huCCL2si2**	11	7	0	
Individual 2	**Control**	0	7	11	0.0001
	**huCCL2si1**	12	4	2	
	**huCCL2si2**	11	2	3	
Individual 3	**Control**	1	7	10	0.01
	**huCCL2si1**	9	6	3	
	**huCCL2si2**	5	9	4	

Three serial tumor sections at three different depths, 50 microns apart were scored in a blinded fashion by three different individuals using a numerical scoring system. 0= No to little invasion. 1= some invasion, 2= high invasion. The scores for each depth were averaged per sample, resulting in 3 scores per sample, and with n=6 tumor samples per group, this resulted in a total 18 scores per group. Statistical analysis was determined by Chi Square test. Statistical analysis was determined by p<0.05. Analyses by individuals are shown.

**Figure 4:**
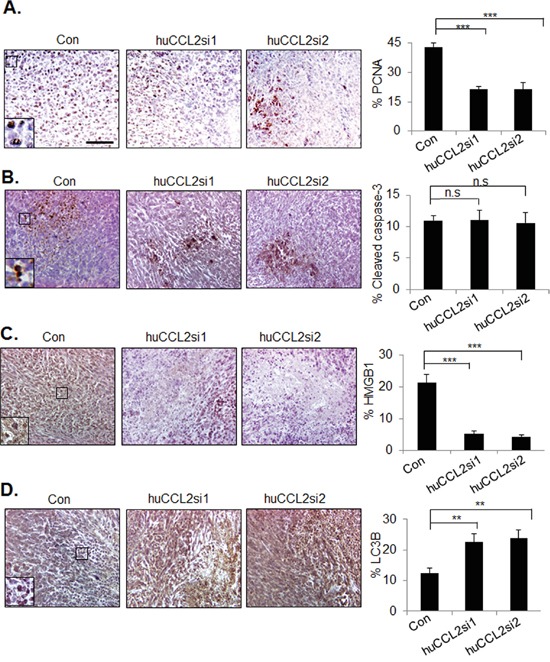
*CCL2* gene silencing enhances necrosis and autophagy in MDA-MB-231 breast tumor xenografts MDA-MB-231 breast tumor xenografts were injected with Ca-TAT peptides complexed to control siRNAs (Con), or CCL2 siRNAs (huCCL2si1 or huCCL2si2), and immunostained for expression of **A.** PCNA, **B.** Cleaved caspase-3, **C.** HMGB1, or **D.** LC3B. Scale=100 microns. Statistical analysis was performed by One Way ANOVA followed by Bonferonni post-hoc analysis. Statistical significance was determined by p-value less than 0.05. *p<0.05, ***p<0.001, n.s=not significant. Mean+SEM is shown. N=6 animals per group.

To further understand the effects of CCL2 silencing on tumor progression, primary breast tumor xenografts were examined for changes in cell proliferation and cell death by immunohistochemistry staining. We observed a significant reduction in PCNA expression from 42.9% with control siRNAs to 21.3% with huCCL2si1 and 21.2% with huCCL2si2. These data indicate decreased tumor cell proliferation (Figure 4A). No changes in cleaved caspase-3 expression were observed in the primary tumors among the experimental groups (Figure 4B). As tumor necrosis was observed by H&E stain, this phenotype was further confirmed by HMGB1 immunostaining. HMGB1 expression was positive in viable tumor cell nuclei. Loss of HMGB1 expression was evident in cells that were absent of hematoxylin staining, indicating loss of intact nuclei. Compared to Ca-TAT/control siRNA treatment, Ca-TAT delivery of either CCL2 siRNAs reduced HMGB1 in the primary tumor by over 75% (Figure 4C), further demonstrating increased necrosis in the primary tumor with CCL2 gene silencing. To identify for possible changes in cellular autophagy, tumors were immunostained for LC3B expression. Compared to Ca-TAT/control siRNA treatment, Ca-TAT delivery of huCCL2si1 or huCCL2si2 increased LC3B expression by over 45% (Figure 4D). Taken together, silencing of CCL2 gene expression results in decreased breast tumor cell proliferation, and increased necrosis and autophagy.

### Ca-TAT/siRNA complexes inhibits the growth of tumor initiating cells

**Figure 5:**
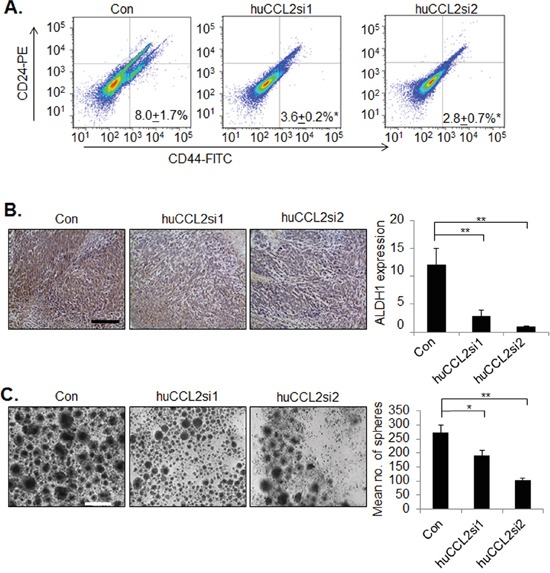
*CCL2* gene silencing in breast cancer cells by Ca-TAT/siRNA complexes inhibits cancer stem cell renewal **A-B.** MDA-MB-231 breast tumor xenografts (N=6 animals per group) were treated with control (Con) or Ca-TAT complexed to CCL2 siRNAs (huCCL2si1, huCCL2si2), and were analyzed for CD24 and CD44 expression by flow cytometry (A), or ALDH1 expression by immunohistochemistry stain (B). ALDH1 expression was quantified by Image J; arbitrary units are shown. Scale bar=100 microns. **C**. MDA-MB-231 cells in culture were transfected with Ca-TAT/siRNA complexes and examined for mammosphere formation. Representative images are shown at passage 4. Scale bar=200 microns. Statistical analysis was performed by One Way ANOVA followed by Bonferonni post-hoc comparisons with Control group. Statistical significance was determined by p-value less than 0.05. *p<0.05, **p<0.01. Mean+SEM is shown.

Tumor initiating cells (also referred to as cancer stem cells or CSCs) contribute to breast cancer recurrence and development of metastatic breast cancer [42]. CSCs resist uptake of conventional chemotherapeutic drugs through a number of mechanisms, including increasing expression of ALDH1 [43, 44]. Thus, ALDH1 is considered a marker for cancer stem cells. Recent studies have shown that CCL2 enhances cancer stem cell renewal of some luminal breast cancer cell lines *in vitro* [45]. However, the relevance of CCL2 expression to cancer stem cell renewal in TNBC is unclear. Therefore, we first determined the effect of CCL2 gene silencing on the numbers of CD24-/CD44+ cells, a well characterized breast cancer stem cell population [46]. By FACs analysis, Ca-TAT delivery of huCCL2si1 or huCCL2si2 decreased the numbers of CD24-/CD44+ cells by over 50% (Figure 5A). By immunohistochemistry analysis of MDA-MB-231 breast tumor xenografts, we observed a significant reduction in ALDH1 expression in CCL2 deficient tumors (Figure 5B). These data indicate that CCL2 knockdown inhibits ALDH1 expression and numbers of CD24-/CD44+ cells in breast tumor xenografts.

We also examined that the effects of CCL2 gene silencing on mammosphere formation *in vitro*. The mammosphere assay, developed by Dontu et al [47] involves culturing of breast epithelial cells under low attachment conditions, which form spheroids in suspension. When these mammospheres were diassociated, these cells grew into new spheroids, which could be measured as an indicator of self–renewal. Dontu et al showed that mammospheres included a mix of progenitor and stem cells. When plated onto a 2D substrate, these spheroids differentiated into ductal and myoepithelial cells [47]. When we cultured MDA-MB-231 cells under low attachment conditions, these cells formed asymmetrical cellular aggregates or “mammospheres,” which could be passaged a minimum of four times, consistent with previous studies [48, 49]. Mammospheres were transfected with Ca-TAT peptides complexed to control or CCL2siRNAs once every passage for up to 4 passages. Compared to the control siRNA treated group, huCCL2si1 or huCCL2si2 significantly decreased the numbers of cellular aggregates formed by MDA-MB-231 breast cancer cells (Figure 5C). Consistently, Ca-TAT delivery of huCCL2si1 and huCCL2si2 resulted in fewer numbers of DCIS.com mammospheres at passage 4 (Supplementary Figure 1). Taken together, these data indicate that CCL2 gene silencing inhibits self-renewal of MDA-MB-231and DCIS.com breast cancer cells.

### Ca-TAT delivery of CCL2 siRNAs inhibit macrophage recruitment in MDA-MB-231 breast tumor xenografts

**Figure 6:**
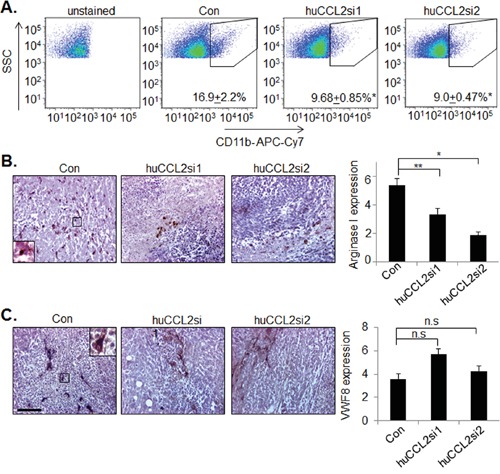
*CCL2* gene silencing in MDA-MB-231 breast tumor xenografts inhibits M2 macrophage recruitment but not tumor angiogenesis MDA-MB-231 breast tumor xenografts were examined for **A.** CD11b expression by flow cytometry, **B.** Arginase I, or **C.** Von Willebrand Factor 8 (VWF8) expression, by immunohistochemistry. Scale bar=100 microns. Expression levels were quantified by Image J; arbitrary units are shown. Statistical analysis was performed by One Way ANOVA followed by Bonferonni post-hoc comparisons with Control group. Statistical significance was determined by p-value less than 0.05. *p<0.05, **p<0.01, n.s= not significant. Mean+SEM is shown. N=6 animals per group

To investigate the mechanisms contributing to the decreased growth and invasion of CCL2 deficient tumors, we examined the possibility that reduced bioavailability of CCL2 protein would inhibit stromal reactivity in the primary tumor. CCL2 promotes angiogenesis [50] and macrophage recruitment [21-23] to primary tumors. Ca-TAT delivery of huCCL2si1 or huCCL2si2 lead to over a 50% reduction in CD11b+ cells in primary tumor xenografts, as determined by flow cytometry (Figure 6A). Imunohistochemistry analysis revealed increased expression of Arginase I expressing cells localized to necrotic areas in control tumors, indicating the presence of M2 macrophages [51, 52]. CCL2 knockdown significantly reduced the number of M2 macrophages localized to necrotic tissues (Figure 6B). CCL2 deficient tumors did not show significant differences in expression of Von Willibrand Factor 8, a blood vessel marker (Figure 6C). In summary, CCL2 gene silencing decreases M2 macrophage recruitment in TNBC xenografts, and does not significantly affect tumor angiogenesis.

**Figure 7:**
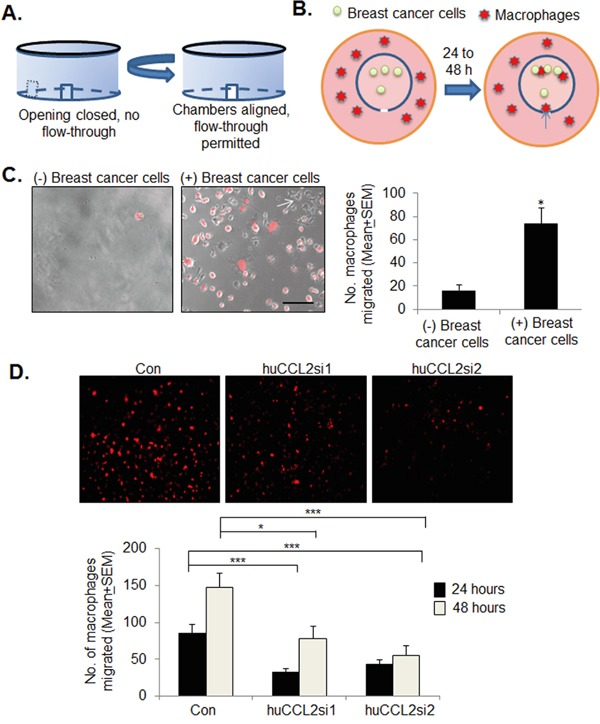
*CCL2* gene silencing inhibits macrophage recruitment to MDA-MB-231 breast cancer 3D cultures **A.** Diagram of MMD function. Dual wall design allows user to control size of flow-through opening by twisting the inner and outer chambers in and out of alignment. **B.** Experimental design: MDA-MB-231 breast cancer cells were embedded in collagen within the MMDs, which were placed in 6 well dishes. mCherry labeled Raw264.7 macrophages were plated outside the devices, and assayed for migration into the MMD over 24-48 hours. **C.** Representative images of Raw 264.7 mcherry macrophage infiltration into collagen alone or collagen embedded with breast cancer cells after 48 hours. Fluorescence images overlayed with phase contrast images are shown. Scale bar=100 microns. Arrow points to unlabeled MDA-MB-231 breast cancer cells. **D.** MDA-MB-231 cells were cultured in 3D collagen in the MMDs, transfected with Ca-TAT/siRNA complexes, and analyzed for macrophage infiltration into the MMD. Macrophage infiltration was quantified by Image J; arbitrary units shown. Statistical analysis was performed by One way ANOVA followed by Bonferonni post-hoc analysis. Statistical significance was determined by p-value less than 0.05. *p<0.05, **p<0.01, ***p<0.0001, n.s=not sigificant. Mean+SEM is shown.

Previous studies have shown that macrophages express CCL2, and have suggested that CCL2 regulates macrophage recruitment to primary breast tumors through a positive feedback loop [22]. To determine the contribution of epithelial specific CCL2 to macrophage recruitment, we used a novel *in vitro* macrophage infiltration assay using a 3D cell culture fluidics device developed in our laboratory, termed the Metastatic Mimetic Device (MMD) [53]. The MMD consists of dual chambers with openings, which fit into one another, and swivels to create a closed system (Figure 7A). The closed system allows for the culture of cells in matrix inside the chamber. Alignment of the chamber openings permits migration of immune cells into the chamber such as macrophages. To determine the contribution of tumoral CCL2 to macrophage recruitment, MDA-MB-231 breast cancer cells were embedded in 3D collagen in the MMD. Ca-TAT/siRNA complexes were transfected into the 3D cultures. Raw 264.7 mcherry cells were plated outside the device, and analyzed for migration into the MMD at 24 and 48 hours (Figure 7B). MDA-MB-231 breast cancer cells embedded in collagen significantly enhanced migration of Raw 264.7 macrophages into the MMD compared to collagen alone, indicating that macrophage infiltration into the MMD was specific to breast cancer cells (Figure 7C). CCL2 knockdown in MDA-MB-231 or DCIS.com cells significantly inhibited the numbers of macrophages migrating into the MMD (Figure 7D, Supplementary Figure 2). These data indicate that epithelial expression of CCL2 is important for regulating macrophage recruitment.

IL-6 and VEGF are two key factors overexpressed in breast tumors [54-56]. VEGF and IL-6 both regulate tumor angiogenesis [57, 58]. IL-6 is also associated with recruitment of myeloid cells and increased cancer cell growth and migration [56, 59, 60]. CCL2 has been shown to regulate expression of IL-6 expression in lung fibroblasts [61] and VEGF in melanoma cells [62]. In addition, studies have shown that treatment of breast tumor xenografts with neutralizing antibodies to CCL2 lead to increased expression of IL-6 and VEGF when anti-CCL2 treatment was interrupted [26]. These studies indicate a functional association between CCL2 expression and VEGF and IL-6. We examined expression of VEGF and IL-6 through immunostaining of MDA-MB-231 tumor xenografts, and did not detect any significant changes in VEGF or IL-6 expression (Supplementary Figure 3A). MDA-MB-231 breast cancer cells transfected with Ca-TAT peptides complexed to CCL2 siRNAs did not show any significant changes in VEGF or IL-6 expression in conditioned medium by ELISA (Supplementary Figure 3B). These data indicate that CCL2 siRNAs do not significantly affect expression of VEGF or IL-6.

## DISCUSSION

The value of CCL2 as a therapeutic target is controversial. Through a novel gene silencing approach, we show that targeting CCL2 expression inhibits tumor progression associated with a reduction in cancer stem cells and M2 macrophages. Previous studies have mainly used neutralizing antibodies to evaluate the role of CCL2 in tumor progression. However, this approach does not affect CCL2 expression. Our studies indicate that targeting CCL2 expression may provide significant therapeutic effects to treat TNBC.

For the first time, we provide proof of concept data that Ca-TAT/siRNA complexes are capable of penetrating breast tumors and inducing gene silencing. Currently, systemic delivery remains the most commonly used method for distributing drugs to primary and secondary tumor tissues, but is highly inefficient, frequently leading to insufficient drug penetration into tissues [63, 64]. These studies underscore a need to identify alternative treatment strategies to enhance both specificity and efficacy of treatment. Localized delivery of drugs to human cancer patients have long been considered a selective and potentially more effective approach for enhancing drug efficacy [65, 66]. In our studies, we show that local delivery of CCL2 siRNAs through Ca-TAT peptide complexes lead to efficient and sustained *CCL2* knockdown in tumor cells, without adversely affecting viability or gene expression in surrounding normal mammary tissue. Localized delivery of Ca-TAT/siRNA complexes to tissues with metastatic lesions could also be effective in therapeutic intervention. Recent studies demonstrated that intra-tracheal delivery of Ca-TAT/pDNA complexes penetrated primary lung cancer [67]. This approach could be used to treat breast cancer patients with existing lung metastases. Further studies must be performed to evaluate the efficacy, pharmacokinetics, and long-term safety of different delivery approaches of Ca-TAT/siRNA complexes for therapeutic intervention of distant metastasis.

One possible concern with local injection is the ability of the Ca-TAT/siRNA complexes to reach their target site before dissociation, due to the weak calcium ionic interactions. A major factor affecting disassociation of the Ca-TAT complexes is pH. Studies of Ca-TAT complexes in the cytosol indicate that low pH may mediate disassociation of the Ca-TAT complexes [68, 69]. At pH 7.4, the particle size of the complexes was shown to be approximately 250 nm irrespective of the CPP used [68]. Particle size increased when the pH was lowered to 4.4, suggesting that an acidic environment reverses the condensing effect of calcium. This pH-induced dissociation was previously reported for DNA/calcium complexes [69, 70]. At the neutral pH, the zeta potential values were positive, indicating that the positively charged CPPs form the bulk of the condensed gene complexes. The net charge on the particles was switched when the pH was lowered, and the gene complexes became anionic or neutral. These data indicate that lowering the environmental pH destabilized the non-covalently condensed pDNA complexes, allowing the pDNA to be exposed or released from the complex [68]. This hypothesis is further supported by other studies of calcium and DNA precipitates, which were shown to dissociate when exposed to similar conditions [70, 71]. The pH in the extracellular space is approximately 7.4 under normal conditions and 7.1 in tumor tissues [72]. Despite the lower pH in the extracellular space of tumor tissues, previous studies showed that Ca-K9 peptide/pDNA complexes significantly attenuated lung tumor growth macroscopically and microscopically when delivered by tail vein injection [68]. These studies indicated that calcium condensed peptide/pDNA complexes were capable of traveling from the tail to the lung before disassociating within the lung tumor cells. Further studies must be performed to more precisely measure the radius from the injection site at disassociation might occur.

CCL2 antibody neutralization inhibited tumor progression in mice bearing 4T1 or MDA-MB-231 breast tumor xenografts [21, 22]. When anti-CCL2 treatment was stopped midway and 4T1 tumors were surgically removed, the anti-CCL2 treated mice showed increased metastasis associated with increased expression of IL-6 and VEGF, compared to control IgG treated mice. From these data, the authors suggested that cessation of CCL2 inhibition may accelerate metastasis by enhancing expression of pro-angiogenic factors [26]. However, it was unclear from that study whether CCL2 antibody neutralization had any significant effects on CCL2 expression levels in the tumors. A phase I clinical trial study on CCL2 antibody neutralization in rheumatoid arthritis patients showed that antibody bound CCL2 complexes were still biologically active and that CCL2 levels were increased in patients [25]. Therefore, it is possible that anti-CCL2 treatment could sequester CCL2 temporarily, but not prevent long-term clearance of CCL2. As a result, CCL2 levels would accumulate once the antibody itself became inactive. In our study, we showed that siRNA delivery effectively reduced CCL2 expression in the primary tumors to levels normally found in normal mammary tissues, and suppressed tumor progression. However, we did not observe any significant changes in VEGF or IL-6 expression in MDA-MB-231 breast tumor xenografts or cells. It is likely that multiple mechanisms regulate expression of these factors. For example, IL-1 and TGF-β are expressed in breast cancer [73], and regulate VEGF and IL-6 expression [74-77]. In summary, our data suggest that directly targeting CCL2 expression would be a more effective means to inhibiting CCL2 biological activity *in vivo*, compared to antibody neutralization. Furthermore, it may not be necessary to eliminate CCL2 expression levels from the tumor to inhibit tumor progression.

In our studies, we found that CCL2 knockdown increased expression of LC3B, indicating cellular autophagy. Autophagy has been associated with cancer cell survival as a means to conserve energy when experiencing metabolic stress [78, 79]. Knockdown of autophagy related genes ATG7 or Beclin1 enhanced necrotic cell death in fibrosarcoma cells [80], indicating that autophagy protects against necrotic cell death. On the other hand, autophagy may also be important for promoting cell death. Autophagic cell death was observed with treatment of ovarian cancer cells with platinum or pituitary cancer cells with cyclosporine [81]. Knockdown of ATG-7 or Beclin1 inhibited necrosis in *Caenorhabditis elegans* [82]. In our studies, it is possible that the increased autophagy in our studies represented a pro-survival mechanism as a reaction to CCL2 knockdown, or functioned to promote cell death. To further characterize the role and mechanisms of autophagy in CCL2 signaling in breast cancer, extensive studies on the autophagy pathway and cell death would be necessary. These studies are beyond the scope of this report, but are of interest in the future.

Our data indicate that CCL2 gene silencing significantly remodeled the microenvironment to suppress tumor progression. In tumors treated with Ca-TAT/control siRNA complexes, central necrosis was surrounded by a large peripheral zone of proliferating tumor cells. Furthermore M2 macrophages were localized to the necrotic areas. These phenotypes in the control tumors are consistent with previous studies showing that tumors may potentially exploit hypoxia induced by necrosis to upregulate angiogenesis and recruit M2 macrophages [83-86]. CCL2 knockdown increased necrosis in the primary tumor. Fewer macrophages were observed in necrotic tissues, and no significant changes in angiogenesis were detected. These studies indicate that decreased CCL2 levels in the primary tumor inhibit the ability of invasive breast tumors to exploit the surrounding host cells. In contrast, antibody neutralization of CCL2 in tumor xenografts enhanced tumor cell apoptosis [21-23]. *CCL2* gene silencing may provide a more selective therapeutic advantage in cases where tumors are resistant to apoptosis [87, 88].

Targeting cancer stem cells represents a promising strategy for therapeutic intervention of recurrent or metastatic breast cancers, which often develop resistance to conventional chemotherapeutics [42, 44]. *In vitro* studies have shown that commonly used therapeutic drugs, such as paclitaxel, selects for survival of breast cancer stem cell populations, possibly contributing to tumor recurrence over time [89, 90]. Cancer stem cells resist the effects of chemotherapeutic drugs through a number of mechanisms including: drug efflux through transporter proteins, pro-survival signaling, increased ALDH1 expression and quiescence [43, 44]. In our studies, we demonstrated that *CCL2* gene silencing in MDA-MB-231 tumor xenografts inhibited ALDH1 expression, and reduced the number of CD24-/CD44+ cells. One likely mechanism is that Ca-TAT/siRNA complexes directly inhibited the growth of CSCs, as indicated by mammosphere assays. Through an indirect mechanism, the reduction in macrophages in CCL2 deficient tumors may have also affected CSC renewal, as M2 macrophages express soluble factors that promote cancer stem cell renewal, tumor growth and invasion [91-93]. Thus, *CCL2* gene silencing via TAT/siRNA complexes inhibit key mechanisms that promote progression of TNBC.

In summary, CCL2 knockdown through delivery of Ca-TAT/siRNA complexes could be an effective treatment strategy for the treatment of invasive breast cancer, particularly when combined with other anti-cancer therapies. Combined with previous studies, our studies indicate that Ca-TAT/siRNA complexes selectively penetrate tumor tissues. TAT peptides are already being tested in the clinic for the treatment of heart disease [94]. Translating Ca-TAT/siRNA complexes as an anti-cancer strategy in the clinic is highly feasible. This low toxicity, high versatility approach supports translation of Ca-TAT/siRNA complexes to the clinic for the treatment of cancer.

## MATERIALS AND METHODS

### Cell culture

MDA-MB-231 cells were obtained from American Tissue Culture Collection. Cancer associated fibroblasts were isolated from invasive breast ductal carcinoma and characterized in previous studies [95, 96]. DCIS.com cells were generously provided by Fariba Behbod, PharmD, Ph.D (University of Kansas Medical Center). Raw 264.7 mcherry cells were generously provided by George Veilhauer, Ph.D (University of Kansas Medical Center, Kansas City, KS). All cell lines were cultured on plastic in DMEM media containing 10% FBS with 0.1% amphotericin, 1% penicillin-streptomycin (cat no. 30-004-CI, Cellgro).

### siRNA/plasmid reagents

Sense and anti-sense oligonucleotides were synthesized and annealed by GE Dharmacon. The following siRNA targeting sequences were designed: enhanced Green Fluorescent Protein (eGFP) as a negative control:5′-GCUGACCCUGAAGUUCAUC-3′ [97], huCCL2si1: 5′-ACCUGCUGUUAUAACUUCA-3′, huCCL2si2: 5′-CAGCAAGUGUCCC AAAGAA-3′.

### Preparation of Ca-TAT complexes

The following formula was used to determine the amount of TAT peptide needed for a specific N/P ratio per μg of DNA or siRNA: μg of TAT = 0.446 *(N/P ratio) + 0.116. For example, complexing 2.346 μg of TAT per 1 μg of DNA or siRNA would yield N/P=5. To prepare complexes, TAT peptides were mixed with siRNA or pDNA in 45 μl sterile deionized water containing: 37.5 mM, 75 mM or 112.5 mM CaCl_2_. The solution was pipetted vigorously for 20 times and incubated on ice for 20 minutes. For 2D and 3D cell culture studies and mammosphere assays, cells were incubated directly with the complexes for 24 hours before media replacement. For *in vivo* studies, 25 μl of 10 % glucose was added to the complexes, and diluted with PBS to a total volume of 100 μl before use.

### 3D cell culture

In each well of a 24 well plate, 100,000 breast cancer cells were embedded in 200 μl rat tail collagen (BD Pharmingen) using methods adapted from previous studies [98]. Briefly, the pH of 2 mg/ml of stock collagen was adjusted by mixing at a 4:1 ratio with setting solution comprised of: 1X EBSS, 75 mM NaOH, and 290 mM NaHCO_3_. Breast cancer cells were detached from the plate by trypnization, quenched, counted by hemocytometer, and pelleted by centrifugation. 100,000 cells were mixed thoroughly with 250 μL collagen solution and pipetted directly in each well, and incubated at 37C for 30 minutes for polymerization of collagen. Cells were incubated in 1 ml of DMEM/10% FBS for 24 hours prior to transfection.

### ELISA

For each well in a 24 well plate, 40,000 cells were seeded in a monolayer or 100,000 cells were seeded in collagen for 24 hours in DMEM/10% FBS. To generate conditioned medium, cells were washed in PBS and incubated in serum free DMEM for 24 hours in 500 μl volume per well. Conditioned media generated from indicated cell lines were subject to ELISA specific to human CCL2 (cat no.900-M31, Peprotech), VEGF (cat no. 900-K10, Peprotech) or IL-6 (cat no. 900-K16, Peprotech). Samples were analyzed according to manufacturer protocol. Reactions were catalyzed using tetramethylbenzidine substrate (cat no. 34028, Pierce) according to manufacturer's instructions. Absorbance was read at OD 450 nM using a BioTek Microplate Reader.

### Animal care and surgery

Female athymic Foxn1nu/nu mice, 6-8 weeks old (Balb/c background) were purchased from Envigo, Inc. Animals were maintained at the University of Kansas Medical Center, in accordance with the Association for Assessment and Accreditation of Laboratory Animal Care (AALAC). All animal experiments were performed at the University of Kansas Medical Center under an approved IACUC. 250,000 human carcinoma associated fibroblasts were embedded with 100,000 MDA-MB-231 cells in 50 μl of collagen rat tail collagen I (BD Pharmingen), using methods previously described [40]. One collagen plugs was transplanted into each mouse, in the #4-5 inguinal mammary glands. When the tumors reached 0.5 cm in diameter, 10 μg (100 μl) of Ca-TAT/control or Ca-TAT/CCL2 siRNA nanoparticles were injected into the primary tumor in 4 different areas of the tumor, using a 27 gauge needle. Each mouse received a total of 3 injections of Ca-TAT/siRNA complexes. Animals were monitored twice weekly for tumor formation by palpation and measurement by caliper. Animals were euthanized 30 days post-transplantation, when control tumors reached maximum allowable tumor size, approximately 1.5 cm^3^ (Figure 2A).

### Tissue embedding

Tissues were fixed in 10 % neutral formalin buffer for 24 hours. Tissues were embedded in wax as described [98]. Briefly, tissues were dehydrated in a series of alcohols: 70, 90, 100% ethanols for 1 hour each. Tissues were further dehydrated in isopropanol for 1 hour, 50:50 ratio of isopropanol: paraffin wax at 60°C for 1 hour and then in wax at 60°C overnight. Tissues were placed into the wax containing molds and allowed to harden at room temperature. Tissues were then processed for histological analysis.

### Histology/immunohistochemistry/immunofluorescence

Wax embedded sections were sectioned at 5 micron thickness onto 1 mm glass slides, dewaxed and rehydrated as described [98]. For H&E stain, slides were incubated in Harris's hematoxylin for 2 minutes and eosin for 2 minutes prior to dehydration and mounting in Cytoseal. For immunostaining, tissue sections were subjected to antigen retrieval through heating in low pressure in sodium citrate buffer pH 6.0 for 2 minutes. Slides were washed in PBS, and endogenous peroxidases were quenched in PBS containing 60 % methanol and 3% H_2_O_2_. Samples were blocked in PBS containing 3% fetal bovine serum for 1 hour, and incubated with primary antibodies (1:100) to: cleaved caspase-3 Asp 175 (cat no. 9579, Cell Signaling Technology), HMGB1 (cat no.6393, Cell Signaling Technology), LC3B (cat no. L10382, Life Technologies), VWF8 (cat no. Ab7356, Millipore), arginase I (cat no. sc20150, Santa Cruz Biotechnology) or VEGF (cat no. sc-152, Santa Cruz Biotechnology) overnight at 4°C. Slides were washed in PBS three times, incubated with secondary rabbit biotinylated antibodies at 1:1000 dilution (cat no. BA-5000, Vector Labs) for 2 hours, conjugated with streptavidin peroxidase (cat no. PK-4000, Vector Labs) and incubated with DAB substrate (cat no. K346711, Dako). For detection of PCNA or IL-6 endogenous mouse immunoglobulins were first incubating with blocking reagents from the Mouse on Mouse kit (cat no. BMK-2202, Vector Laboratories), according to commercial protocol. Slides were incubated with PCNA antibodies (cat no. sc25280, Santa Cruz Biotechnology) or IL-6 antibodies (cat no. MAB-2061m RnD Systems) at a 1:100 for 1 hour and then incubated with secondary mouse biotinylated antibodies from the MOM kit at a 1:250 dilution for 1 hour. For detection of CD24, slides were incubated with antibodies at a 1:100 (cat no. 561777 BD Pharmingen), and detected with secondary rat-biotinylated antibodies (cat no.BA-9401, Vector Laboratories) at a 1:1000 dilution. Sections were counterstained with Harris' hematoxylin for 1 minute, dehydrated and mounted with Cytoseal. Because tumor sections were of various sizes, it was determined that 4 fields could be captured consistently among tumor samples, while enabling a good representation of the tumor. Therefore, four random fields per section, with 2 sections per sample, were captured at 10 x magnification using a Motic AE 31 microscope with Infinity2-1c color digital camera. DAB staining was quantified by pixel density analysis, normalized to total tumor area, using an Image J software protocol (NIH) described in previous studies [98-101].

For immunofluorescence, sections were incubated at 1:100 dilution of antibodies to Cytokeratin 5 (CK5, cat no. XM-26, ThermoFisher) and Calsequestrin (cat no. SC-28274, Santa Cruz Biotechnologies overnight at at 4°C, conjugated with anti-rabbit-Alexa-568 and anti-mouse-Alexa-488 at a 1:500 dilution for 2 hours. Sections were washed in PBS, counterstained with DAPI and mounted with PBS/glycerol.

### Quantification of tumor necrosis

To quantify the extent of necrosis in breast tumor xenografts, tumor tissues were sectioned at 5 different depths, approximately 50 microns apart. 3 sections from each depth were placed on a slide and stained by H&E. Sections were imaged at 4x magnification, at 2-3 fields per section to capture the whole tissue section. Software analysis for necrotic areas in breast tissues was performed using methods adapted from previous studies [96]. Images were first imported into Adobe Photoshop. Color and exposure of images were normalized using auto-contrast. Necrotic areas were selected using a lasso tool, copied to a new window and saved a separate file. Images were opened in Image J software (NIH), and converted to grey scale. Background pixels resulting from luminosity of brightfield images were removed by threshold adjustment. Images were the subject to particle analysis. Necrotic areas and total areas were expressed as particle area values of arbitrary units. Values representing necrotic areas were normalized to values representing total tissue section.

### Scoring of tumor invasion

To quantify the extent of tumor invasion into muscle tissue, tumor tissues were sectioned at 3 different depths approximately 50 microns apart. Three serial sections from each depth were placed on each slide and stained by H&E. The muscle tissue was distinguished by a striated appearance that was strongly positive for eosin stain. Each section from each sample was scored for extent of invasion by analysis of slides at 4x and 10x magnification. Three different individuals in blinded studies used a numerical scoring system. 0 indicated no to low invasion, characterized by no or a few tumor viable cells present in muscle tissue, or the presence of necrotic tumor cells in muscle tissue; the border between muscle and tumor tissue was well defined. 1 indicated some tumor cell invasion, characterized by viable tumor cells present in muscle tissue; the border between muscle and tumor tissue was less defined. 2 indicated high invasion characterized by extensive number of tumor cells in muscle tissue; tumor was embedded in muscle, and the border between muscle tissue and tumor undefined.

### Quantitation of lung metastases

Metastatic nodules in lung tissues were detected and quantified using hematoxylin staining approach of lung tissues, as previously described in previous studies [99]. Briefly, tissues were dehydrated a series of alcohols: 70, 90, 100% ethanols for 1 hour each and cleared in xylene overnight. Tissues were rehydrated in decreasing series of ethanols, flushed with running tap water for 15 minutes and then stained with Gill's hematoxylin for 10 minutes. Lung tissues were flushed with water for 5 minutes, de-stained in 1 % HCl for 20 minutes, and then incubated in tap water overnight. Tissues were partially dehydrated in 70% ethanol for 1 hour. Metastatic lesions throughout the lung tissue were visually identified by hematoxylin staining as round shaped nodules under brightfield/phase contrast microscopy using a Motic AE31 inverted microscope (Motic AE31). Metastatic nodules were manually scored in the lung tissues. The presence of metastases was confirmed by paraffin embedding of whole tissue, and H&E stain of lung sections.

### Flow cytometry

For adherent cells in culture, cells were first detached from plastic by washing in PBS twice, and incubation in 3 mM EDTA at 37°C for 10-15 minutes. Cells were washed with 10 ml of complete medium twice, fixed in 10 % neutral formalin buffer for 10 minutes at room temperature and washed with PBS twice to remove traces of formalin. For tumor tissues, tissues were washed in PBS, and digested into single cell suspensions with collagenase I and trypsin for 4 hours at 37°C, as described in previous studies [21, 102-104]. Tumor cell suspensions were fixed in 10% NBF for overnight at 4C, and then washed with PBS twice to remove traces of formalin. Cells were permeabilized with 0.1 % Triton-X 100 in a 37°C water bath for 15 minutes, and washed in PBS twice. Samples were incubated with the following antibodies at 1: 50 dilution, overnight on ice in PBS containing 2% BSA: CD24- PE (cat no.555428, BD Pharmingen), CD11b-APC-Cy7 (cat. no 557657, BD Pharmingen), murine CCL2 (cat no. 1784, Santa Cruz Biotechnology), human CCL2 (cat no. sc1304, Santa Cruz Biotechnology) Ki67 (cat no. Sc15402, Santa Cruz Biotechnology), HMGB1 (cat no. 6893, Cell Signaling Technology) or LC3B (cat no. L10382, Invitrogen). Samples were incubated with anti-Fsp1 at a dilution of 1:3 (cat no. ab75550, Abcam). Murine CCL2 and human CCL2 were detected by secondary goat antibodies conjugated to Alexa-488 (1:500) in PBS for 1 hour on ice, covered in foil. Fsp1, Ki67, HMGB1, LC3B were detected by secondary rabbit antibodies conjugated to Alexa-647 at a 1:500 dilution on ice for 1 hour. Cells were washed with PBS three times prior to analysis. Samples were analyzed on a LSRII Flow cytometer, normalized to secondary antibody only controls.

### Mammosphere assay

30,000 cells were seeded in low attachment 6 well plates (Corning) in 3 ml of DMEM/10% FBS. 48 hours after plating, cells were transfected with Ca-TAT complexed to 3 ug of control, huCCL2si1 or huCCLsi2 siRNAs. After an additional 5 days in culture, mammospheres were collected in 15 ml conical tubes, pelleted, and disassociated using 20 mM Trypsin/2 mm EDTA solution for 7 minutes at 37C. Cells were pelleted and replated for 48 hours before re-transfection with Ca-TAT/siRNA complexes. Images were captured using the EVOS FL auto every 7 days of plating. Mammospheres were counted using Image J software.

### 3D macrophage infiltration assay

MMD fluidic devices were fabricated by MetaBioscience LLC (Overland Park, KS). For each chamber device, 100,000 breast cancer cells were embedded in 250 μl rat tail collagen (BD Pharmingen), as described for 3D cultures. Devices were placed in 6 well dishes. 1 ml of DMEM/10% FBS was pipetted into the device and incubated overnight. After overnight incubation of the devices, 500,000 Raw 264.7 mcherry cells were counted and re-suspended into 2.5 ml of DMEM/10% FBS for each device. Devices were twisted open and cells were pipetted into each well, outside of the device. Devices were imaged daily at 10x magnification using an EVOS FL auto imaging system (Invitrogen) for up to 48 hours. The number of macrophages were measured by quantification of fluorescence using methods previously described [98].

### Statistical analysis

All experiments were repeated a minimum of three times. Data are expressed as mean+ standard error of the mean (SEM) Statistical analysis was determined using Two-Tailed T-test or ANOVA with Bonferonni's post-hoc comparisons using Graphpad Software. Significance was determined by p<0.05. *p<0.05, **p<0.01, ***p<0.0001, n.s=not significant or p>0.05.

## SUPPLEMENTARY MATERIALS FIGURES


