# Supplementary material for: Targeted gene silencing of CCL2 inhibits triple negative breast cancer progression by blocking cancer stem cell renewal and M2 macrophage recruitment
Source: Oncotarget. 2016 Jun 7;7(31):49349–67. doi: 10.18632/oncotarget.9885 (PMC5226513; doi:10.18632/oncotarget.9885)
Supplement: Supplementary file 1 [file oncotarget-07-49349-s001.pdf]

Targeted gene silencing of CCL2 inhibits triple negative breast cancer progression by blocking cancer stem cell renewal and M2 macrophage recruitment

SUPPLEMENTARY FIGURES

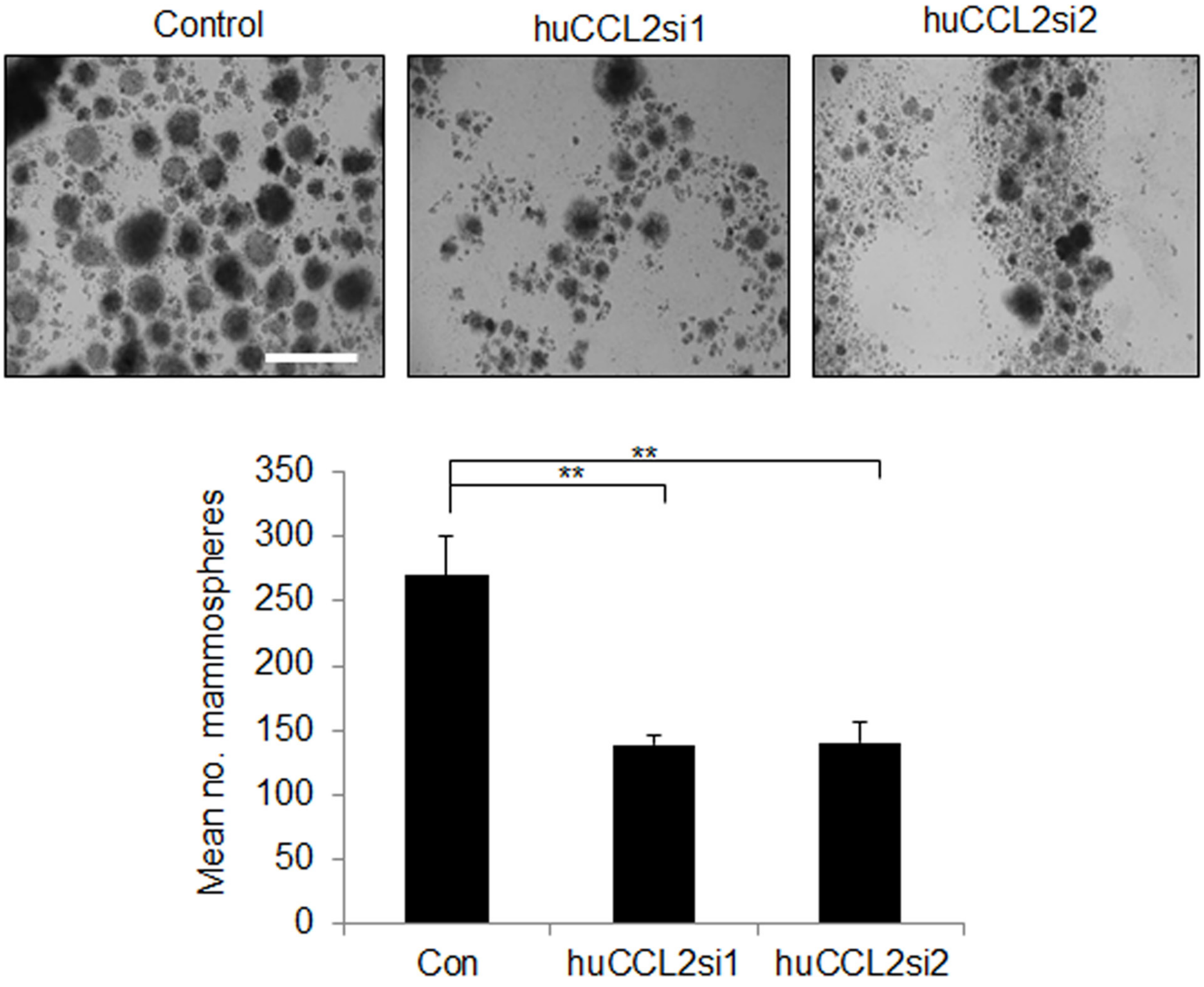

Supplementary Figure S1: Ca-TAT mediated delivery of CCL2 siRNAs decreases the numbers of DCIS.com mammospheres. DCIS.com cells were transfected with Ca-TAT/siRNA complexes and analyzed for mammosphere formation for 4 passages. Representative images of mammospheres at passage 4 are shown. Scale bar=200 microns. Mammospheres were quantified by Image J analysis. Mean number of mammsopheres at passage 4 is shown. Statistical analysis was performed using One Way ANOVA with Bonferonni post-hoc analysis. Statistical significance was determined by p-value less than 0.05. **$p<0.01$. Mean±SEM is shown.

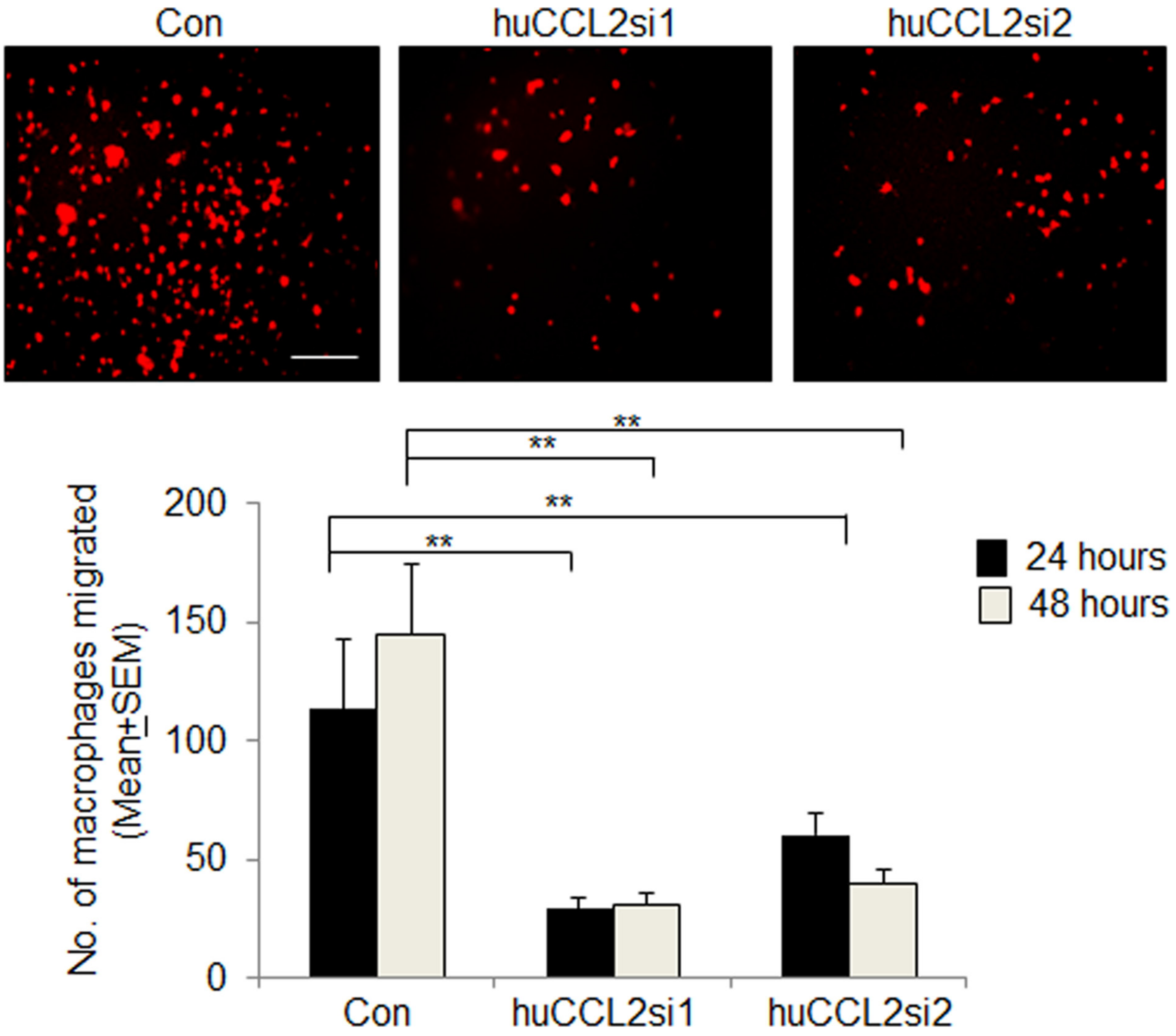

Supplementary Figure S2: CCL2 gene silencing in DCIS.com breast cancer cells inhibits macrophage infiltration into the MMD. DCIS.com cells were cultured in 3D collagen in the MMDs, transfected with Ca-TAT/siRNA complexes, and analyzed for macrophage infiltration into the MMD. Scale bar=100 microns. Macrophage infiltration was quantified by Image J; arbitrary units shown. Statistical analysis was performed by One way ANOVA followed by Bonferonni post-hoc analysis. Statistical significance was determined by p-value less than 0.05. **$p<0.01$. Mean+SEM is shown.

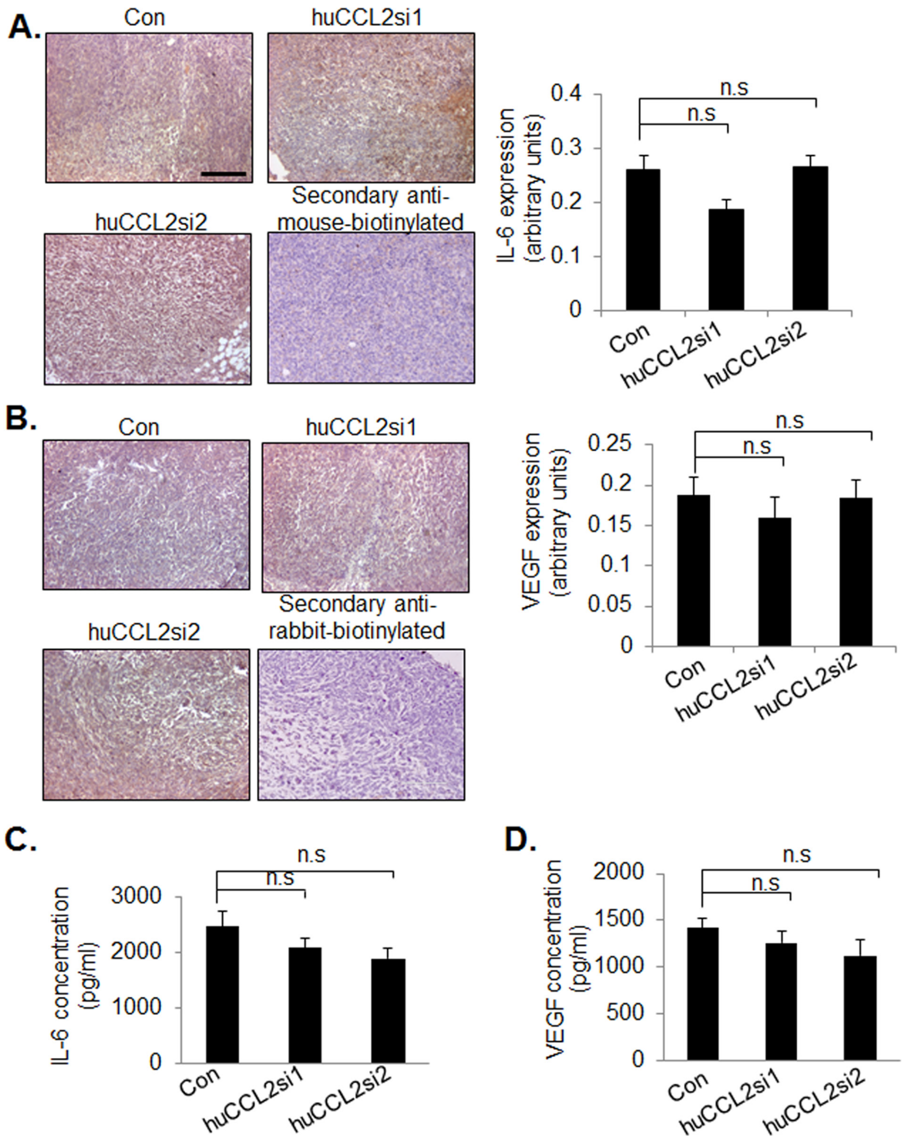

Supplementary Figure S3: Ca-TAT delivery of CCL2 siRNAs does not significantly affect VEGF or IL-6 expression in MDA-MB-231 breast tumor xenografts or cells. A-B. MDA-MB-231 breast tumor xenografts were immunostained for protein expression of IL-6 (A) or VEGF (B). Scale bar= 200 microns. Protein expression was quantified using Image J software. **C-D.** MDA-MB-231 breast cancer cells were embedded in 3D collagen, transfected with Ca-TAT peptides complexed to siRNAs, and analyzed for expression of IL-6 (C) or VEGF (D) in conditioned media by ELISA. Statistical analysis was performed by One way ANOVA followed by Bonferonni post-hoc analysis. Statistical significance was determined by p-value less than 0.05. n.s=not significant. Mean±SEM is shown.
